# Patient–Physician Discordance and Unmet Needs in Rheumatoid Arthritis: A Network Analysis of Clinical and Quality-of-Life Domains

**DOI:** 10.3390/jcm15062152

**Published:** 2026-03-12

**Authors:** Selçuk Akan, Mustafa Uğurlu, Yüksel Maraş, Kevser Orhan, Samet Çevik, Görkem Karakaş Uğurlu, Ebru Atalar

**Affiliations:** 1 Internal Medicine Department, Physical Medicine and Rehabilitation Hospital, Ankara City Hospital, Ankara 06800, Turkey; 2 Psychiatry Department, Ankara City Hospital, Yıldırım Beyazıt University, Ankara 06800, Turkey; dr_ugurlu@yahoo.com (M.U.); dr_gorkem@yahoo.com (G.K.U.); 3 Department of Rheumatology, Ankara Bilkent City Hospital, Health Sciences University, Ankara 06800, Turkey; ymaras@hotmail.com; 4 Department of Physical Medicine and Rehabilitation and Rheumatology, Ankara City Hospital, Yıldırım Beyazıt University, Ankara 06800, Turkey; kevserorhan.md@gmail.com; 5 Family Medicine Department, Ankara Bilkent City Hospital, Ankara 06800, Turkey; samcevik@gmail.com; 6 Department of Rheumatology, Ankara Bilkent City Hospital, Ankara 06800, Turkey; atalarebrudr@yahoo.com

**Keywords:** rheumatoid arthritis, unmet needs, patient–physician discordance, network analysis, pain, quality of life, patient-reported outcomes

## Abstract

**Background:** Despite the widespread implementation of treat-to-target strategies and modern disease-modifying antirheumatic drugs, a substantial proportion of patients with rheumatoid arthritis (RA) continue to report unmet needs (UNs), defined as a mismatch between patient expectations and symptom burden on the one hand and outcomes achieved with current care on the other. Patient–physician discordance in global assessments may reflect multidimensional influences, including pain mechanisms, psychosocial factors, functional impairment, and communication gaps, extending beyond inflammatory disease activity. **Methods:** In this cross-sectional study, 133 patients with RA and 57 healthy controls were included. UNs were operationalized as the signed difference between patient global assessment and physician global assessment (ΔPGA–PhGA). Clinical variables, patient-reported outcomes, and Short Form-36 (SF-36) domains were incorporated into two regularized partial correlation network models estimated using the extended Bayesian information criterion graphical least absolute shrinkage and selection operator (EBICglasso). Node centrality indices (strength, signed strength, betweenness, and closeness) were calculated. Network stability was evaluated using 2000 bootstrap resamples and correlation stability (CS) coefficients. **Results:** In the clinical network, pain intensity demonstrated the highest strength centrality and the strongest direct association with UNs. In contrast, Disease Activity Score in 28 joints with C-reactive protein (DAS28-CRP) showed no direct association with UNs after accounting for shared variance. In the SF-36-based quality-of-life network, UNs exhibited inverse associations, particularly with perceived health change and role–emotional functioning. Stability analyses indicated acceptable to good robustness (clinical network: CS = 0.59 for edge weights and 0.44 for strength; SF-36 network: CS = 0.59), supporting the reliability of the estimated network structures. **Conclusions:** UNs in RA are not solely determined by inflammatory disease activity but are embedded within interconnected clinical and psychosocial domains. Pain occupies a structurally central position in the clinical network, whereas perceived health change and emotional role limitations characterize the quality-of-life context of UNs. These findings underscore the importance of multidimensional and patient-centered assessment strategies in RA management.

## 1. Introduction

Rheumatoid arthritis (RA) is a chronic, systemic inflammatory disease characterized by persistent synovitis, progressive joint damage, pain, fatigue, and functional impairment, ultimately leading to a substantial deterioration in quality of life [1]. Despite major advances in therapeutic strategies and the widespread implementation of treat-to-target approaches, a considerable proportion of patients continue to experience symptoms that remain insufficiently controlled, even when clinical disease activity targets appear to be achieved [2,3].

Current RA management relies on disease-modifying antirheumatic drugs (DMARDs), including conventional synthetic (csDMARDs), biologic (bDMARDs), and targeted synthetic DMARDs (tsDMARDs). These treatment classes differ in their mechanisms of action, therapeutic profiles, and impact on patient-reported outcomes [4]. Although these therapies have substantially improved inflammatory control and structural outcomes in RA, complete alignment between clinical targets and patient experience is not always achieved. Nevertheless, discordance between physician-assessed disease control and patient-perceived disease burden remains a frequent and clinically relevant challenge in routine practice.

Patient global assessment (PGA) and physician global assessment (PhGA) constitute integral components of composite disease activity indices. Whereas PhGA predominantly reflects clinical examination findings and laboratory parameters, PGA captures subjective experiences such as pain, fatigue, and the overall impact of disease on daily functioning. Discordance between PGA and PhGA has consistently been associated with poorer functional outcomes, impaired quality of life, and reduced treatment satisfaction [5,6,7].

UNs in RA have been conceptualized as discrepancies between patients’ expectations, perceived symptom burden, and achieved clinical outcomes [8]. PGA–PhGA discordance has therefore been proposed as a pragmatic proxy for UN; however, it may also reflect communication gaps and psychosocial dimensions that are not fully captured by conventional disease activity indices [6,8].

RA is inherently multidimensional, with pain, functional disability, psychological distress, and health-related quality of life closely interconnected [9,10]. Traditional analytical approaches often evaluate these domains in isolation, potentially overlooking their conditional interdependencies. Network analysis enables the simultaneous modeling of interrelated variables and provides a systems-level perspective on complex clinical phenomena [11].

The aim of the present study was to investigate UNs in RA, operationalized as the signed difference between PGA and PhGA (ΔPGA–PhGA), and to explore their structural relationships within interconnected clinical and quality-of-life domains using network analysis.

## 2. Materials and Methods

### 2.1. Study Population and Study Design

This cross-sectional observational study was conducted in patients with rheumatoid arthritis (RA) who were followed at Ankara Bilkent City Hospital between January 2021 and January 2024.

Healthy controls were included to provide a comparative reference for functional status and health-related quality-of-life measures, thereby enabling contextual interpretation of the burden observed in patients with RA. Healthy controls were used exclusively for descriptive and comparative analyses and were not included in network models.

Patient global assessment (PGA) and physician global assessment (PhGA) were collected only from patients with RA, as these measures are anchored to the clinical evaluation of active disease and require physician judgment regarding disease severity. Accordingly, calculation of patient–physician discordance (UN score) was not applicable to healthy controls.

### 2.2. Participants

A total of 133 consecutive patients aged ≥18 years who fulfilled the 2010 ACR/EULAR classification criteria for RA were enrolled in this cross-sectional study [8]. Eligible patients presenting to the internal medicine or rheumatology outpatient clinics were consecutively included after meeting the inclusion criteria and providing informed consent.

Clinical assessments were performed by two rheumatologists responsible for routine follow-up of their patients and one internal medicine specialist with rheumatology training.

In addition, 57 age- and sex-comparable healthy controls were recruited from hospital staff, patients’ relatives, and community volunteers. Controls were selected to achieve comparable group-level age and sex distribution; individual matching was not performed. Healthy controls had no history of chronic inflammatory, autoimmune, or psychiatric disorders. They were included solely as a reference group for functional status and quality-of-life comparisons and were not considered in UN or network analyses.

Exclusion criteria for all participants were

1. Presence of other systemic autoimmune or inflammatory diseases;
2. Acute infection or malignancy;
3. Severe psychiatric illness;
4. Incomplete clinical or questionnaire data.

### 2.3. Clinical and Patient-Reported Outcome Measures

All participants underwent standardized clinical, psychosocial, and quality-of-life assessments conducted by trained investigators. Questionnaires were completed with physician assistance to ensure accurate comprehension and responses reflecting current clinical and psychological status.

Pain intensity was assessed using a 10 cm (100 mm) visual analog scale (VAS). Disease activity was evaluated using the 28-joint Disease Activity Score calculated with C-reactive protein (DAS28-CRP), based on routine joint examination.

Functional disability was measured using the Health Assessment Questionnaire (HAQ) raw total score. The untransformed raw sum of all 24 HAQ item responses (range 0–24) was used for statistical analyses rather than the conventional 0–3 Disability Index to preserve score variability and avoid information loss due to rescaling. RA-specific quality of life was assessed using the Rheumatoid Arthritis Quality of Life questionnaire (RAQoL).

Psychological distress was evaluated using the Depression Anxiety Stress Scale-42 (DASS-42) total score. Generic health-related quality of life was assessed using the Short Form-36 (SF-36), comprising eight domains (physical functioning, role physical, role emotional, vitality, mental health, social functioning, bodily pain, and general health perception) and a single-item measure of perceived health change over the preceding year.

PGA and PhGA were recorded using a 10 cm (100 mm) VAS. Patients were asked, *“Considering your symptoms, daily functioning, and overall well-being, how would you rate your rheumatoid arthritis activity today?”* Physicians were asked, *“Considering clinical findings, laboratory results, and overall disease evaluation, how would you rate this patient’s rheumatoid arthritis activity today?”* Higher scores indicated greater perceived disease activity.

Demographic variables included age, sex, marital status, personal history of psychiatric illness, and family history of psychiatric disorders.

### 2.4. Definition of PGA–PhGA Discordance

Patient–physician discordance was defined as the directional (signed) difference between PGA and PhGA scores (ΔPGA–PhGA), allowing positive values when patient ratings exceeded physician ratings and negative values in the opposite case. This continuous variable served as the primary operational definition of UNs in all statistical and network analyses.

For descriptive purposes only, a data-driven threshold based on the median ΔPGA–PhGA value of the cohort (≥2 units) was used to identify patients with relatively higher UN. Median-based categorization is considered a pragmatic approach in exploratory cross-sectional analyses when no universally established clinical cut-off exists and when stratification is descriptive rather than inferential [9].

### 2.5. Statistical Analysis and Network Analysis

All statistical analyses were performed using IBM SPSS Statistics version 25 (IBM Corp., Armonk, NY, USA). Continuous variables were assessed for normality using the Shapiro–Wilk test and visual inspection of histograms and Q–Q plots. As distributional assumptions were met, continuous variables were summarized as mean ± standard deviation and compared between RA patients and healthy controls using independent-samples t-tests. Effect sizes were calculated using Cohen’s d. Categorical variables were summarized as frequencies and percentages and compared using the chi-square test.

UNs (ΔPGA–PhGA) were analyzed as a continuous variable in all inferential and network analyses. Statistical significance was defined as a two-sided *p* value < 0.05.

Network analyses were conducted in R (version 4.3.2) using the qgraph and bootnet packages. Two regularized partial correlation networks were estimated using the extended Bayesian information criterion graphical least absolute shrinkage and selection operator (EBICglasso) method.

The clinical network included

- VAS pain;
- DAS28-CRP;
- HAQ raw total score (0–24);
- DASS-42 total score;
- RAQoL score;
- UNs (ΔPGA–PhGA).

The quality-of-life network included all SF-36 domains and UNs. Node-level centrality indices—strength, signed strength, closeness, and betweenness—were calculated to assess relative importance within the network. Strength represents the sum of absolute edge weights connected to a node, whereas signed strength accounts for directionality. Network accuracy and stability were evaluated using nonparametric and case-dropping bootstrap procedures with 2000 resamples. Correlation stability (CS) coefficients were calculated to assess robustness; values >0.25 were considered acceptable and >0.50 good, in accordance with established methodological recommendations [10].

The healthy control group was included solely in descriptive and comparative analyses. All network analyses were performed exclusively within the RA cohort.

### 2.6. Ethical Approval

The study was conducted in accordance with the Declaration of Helsinki and approved by the Bilkent City Hospital Ethics Committee (E2-21-556; 2 June 2021). Patients enrolled after ethics approval were included prospectively following written informed consent. Data obtained prior to ethics approval were derived from routine clinical assessments and analyzed retrospectively after approval was granted. Written informed consent was obtained from all participants before inclusion. A blank informed consent form is available for editorial review.

## 3. Results

### 3.1. Participant Characteristics and Group Comparisons

A total of 133 patients with rheumatoid arthritis (RA) and 57 healthy controls (HCs) were included. Sex distribution did not differ significantly between groups (RA: 74.4% female; HC: 61.2% female; χ^2^, *p* = 0.082). The proportion of married participants was higher in the RA group (*p* < 0.001), whereas divorced or widowed status did not differ significantly. Personal (12.9% vs. 16.3%) and family history (3.8% vs. 6.1%) of psychiatric disorders were comparable between groups (both *p* > 0.05).

RA patients were significantly older than controls (51.77 ± 12.24 vs. 44.84 ± 13.98 years; *p* = 0.002). Functional disability (HAQ raw score) and RA-specific quality-of-life impairment (RAQoL) were markedly higher in RA (both *p* < 0.001), with large effect sizes (Cohen’s d = −1.08 and −1.18, respectively). No significant difference was observed in total DASS-42 scores (*p* = 0.768).

Across all Short Form-36 (SF-36) domains, RA patients demonstrated significantly lower scores than controls (all *p* values between <0.001 and 0.009), indicating broadly impaired health-related quality of life. The largest between-group differences were observed for bodily pain and general health perception (Cohen’s d = 0.96 and 0.92, respectively). Detailed results are presented in Table 1A.

UN (ΔPGA–PhGA) was calculated exclusively in the RA cohort. The mean value was 2.27 ± 2.05, with a median of 2.00 (IQR: 1.00–4.00) and a range from −1 to 8. Positive values predominated, indicating that patient-rated disease burden generally exceeded physician assessment. A minority of patients exhibited negative values, reflecting higher physician ratings.

Using the median threshold (≥2 units), 81 patients (60.9%) were classified as having higher UN. Descriptive statistics are summarized in Table 1B.

Values are presented as mean ± standard deviation (SD) unless otherwise indicated. Group comparisons were performed using independent samples *t*-tests for continuous variables and chi-square tests for categorical variables. Effect sizes were calculated using Cohen’s d.

### 3.2. Network Analysis: Quality-of-Life Network (SF-36)

In the SF-36-based network, the highest strength centrality values were observed for Role–Physical (1.13), Vitality (1.02), and Social Functioning (0.95), followed by Mental Health (0.91), Bodily Pain (0.89), Physical Functioning (0.88), and General Health Perception (0.86). Role–Emotional (0.81) and Health Change (0.79) showed moderate centrality.

The UN node exhibited comparatively low strength (0.33) and negative signed strength (−0.33), indicating inverse associations with quality-of-life domains. Thus, higher UNs were consistently associated with lower SF-36 scores.

**Figure 1:**
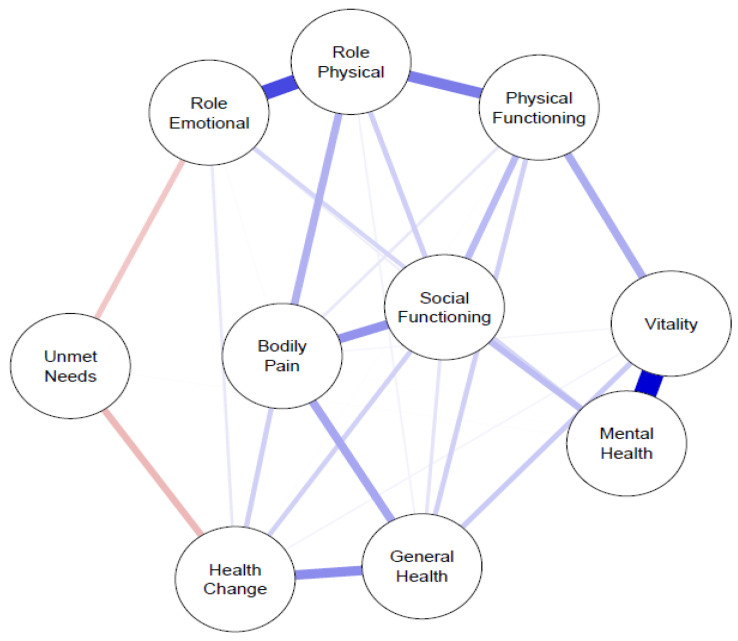
Regularized partial correlation network of SF-36 domains and unmet needs in patients with rheumatoid arthritis.

Betweenness centrality was highest for Role–Physical and General Health Perception (0.19 each), whereas UNs demonstrated minimal betweenness (0.03), suggesting a limited intermediary role. Closeness centrality values were relatively homogeneous; UNs showed the lowest closeness (0.09). Centrality indices are detailed in Table 2, and the network structure is illustrated in Figure 1.

Values represent node-level centrality indices derived from the regularized partial correlation network estimated using the EBICglasso method. Strength reflects the sum of absolute edge weights connected to each node, whereas signed strength accounts for the direction of associations. Betweenness indicates the extent to which a node lies on the shortest paths between other nodes, and closeness reflects the average inverse shortest path length to all other nodes. Higher values indicate greater relative centrality within the network.

The network was estimated using the extended Bayesian information criterion graphical least absolute shrinkage and selection operator (EBICglasso). Nodes represent SF-36 domains and UNs (ΔPGA–PhGA). Edges represent regularized partial correlations after controlling for all other variables in the network. Blue edges indicate positive associations, whereas pink edges indicate negative associations. Edge thickness corresponds to the magnitude (absolute value) of the partial correlation coefficient. The analysis was performed exclusively within the rheumatoid arthritis cohort.

### 3.3. Network Analysis: Clinical Network

Within the clinical network, the highest strength values were observed for pain intensity (VAS; 1.33) and RAQoL (1.32). UNs also demonstrated relatively high strength (1.13), indicating substantial connectivity with other clinical variables. Lower strength values were observed for DAS28-CRP (0.78), HAQ raw score (0.73), and DASS-42 (0.51).

**Figure 2:**
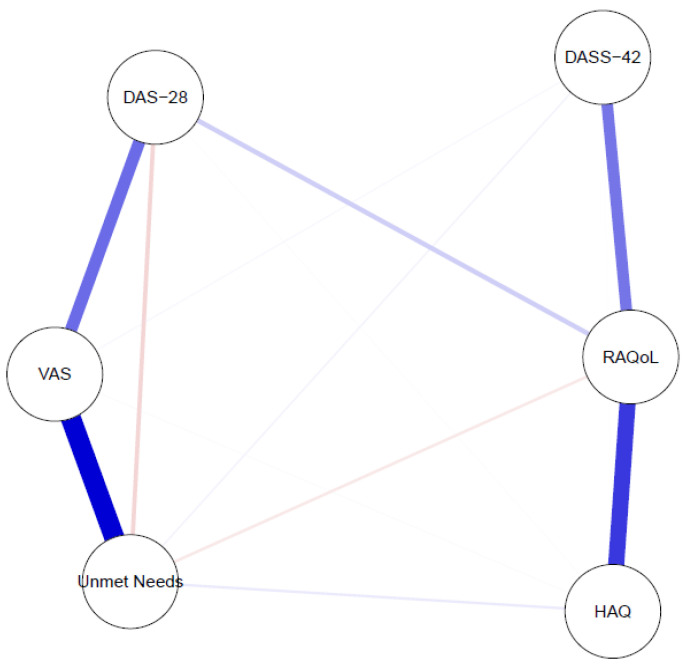
Regularized partial correlation network of clinical variables and unmet needs in patients with rheumatoid arthritis.

All nodes showed positive signed strength values; UNs had a signed strength of 0.71. Betweenness centrality was highest for RAQoL (0.70) and DAS28-CRP (0.60), followed by VAS (0.40). In contrast, UNs, HAQ, and DASS-42 exhibited zero betweenness, suggesting that they did not function as intermediary bridge nodes. Closeness centrality was highest for RAQoL and DAS28-CRP (0.18), while UNs and DASS-42 showed similar moderate values (0.13). Detailed metrics are provided in Table 3, and the graphical network is shown in Figure 2.

Importantly, pain intensity demonstrated the strongest direct association with UNs, whereas DAS28-CRP was not directly connected after controlling for shared variance.

Centrality indices were calculated from the EBICglasso-based regularized partial correlation network. Strength represents the total connectivity of a node, while signed strength incorporates the directionality of associations. Betweenness quantifies intermediary influence between nodes, and closeness reflects network integration. Higher centrality values indicate greater structural importance within the clinical network.

The network was estimated using EBICglasso regularization. Nodes represent pain intensity (VAS), Disease Activity Score in 28 joints with C-reactive protein (DAS28-CRP), functional disability (HAQ raw score), psychological distress (DASS-42 total score), rheumatoid arthritis-specific quality of life (RAQoL), and UNs (ΔPGA–PhGA). Edges denote regularized partial correlations conditioned on all other variables in the network. Blue edges represent positive associations, and pink edges represent negative associations. Edge thickness reflects the strength of the association. The network includes only patients with rheumatoid arthritis.

### 3.4. Network Stability and Robustness

Network stability was evaluated using nonparametric and case-dropping bootstrap procedures (2000 resamples). Correlation stability (CS) coefficients exceeded recommended minimum thresholds (CS > 0.25) in both models. Simulation studies suggest that CS coefficients above 0.50 indicate good stability for the interpretation of centrality indices in psychological and clinical network models [11].

In the clinical network, CS coefficients were 0.59 for edge weights and 0.44 for strength centrality, indicating good stability. In the SF-36 network, CS coefficients were 0.59 for both edge weights and strength, demonstrating robust estimation despite the larger node set. These findings support cautious interpretation of centrality indices within the exploratory framework.

### 3.5. Overall Network Findings

Distinct structural patterns emerged across domains. In the clinical network, UNs were closely linked to pain intensity and RA-specific quality-of-life impairment. In contrast, within the SF-36 network, UNs demonstrated inverse associations primarily with perceived health change and role–emotional functioning, suggesting a more indirect relationship with generic quality-of-life domains.

## 4. Discussion

Despite major advances in rheumatoid arthritis (RA) management through treat-to-target strategies and modern disease-modifying antirheumatic drugs, a substantial proportion of patients continue to report residual symptoms and UNs [1,2,3,4]. Achieving low disease activity or remission according to composite indices does not necessarily translate into optimal patient-perceived well-being [9,10]. In the present study, UNs were operationalized as the signed difference between patient and physician global assessments (ΔPGA–PhGA), reflecting discordance between perceived disease burden and physician-rated activity rather than specific unmet therapeutic interventions. Using a network analysis framework, we examined how this discordance is embedded within interconnected clinical and quality-of-life domains.

Within the clinical network, pain intensity emerged as the most central variable and showed the strongest direct association with UNs. This pattern suggests that pain is tightly interwoven with multiple clinical domains and may have broader downstream implications for patient experience. Consistent with prior work, higher patient global ratings and dissatisfaction appear to be driven predominantly by pain rather than objective inflammatory measures [10,12]. Notably, DAS28-CRP was not directly linked to UNs after accounting for shared variance, supporting concerns that composite disease activity indices may not fully capture patient-relevant burden [3,5]. Collectively, these findings highlight the persistent role of residual pain in shaping UNs, even when inflammation appears controlled.

The observed dissociation between inflammatory activity and UNs may, in part, reflect non-inflammatory pain mechanisms. Central sensitization and fibromyalgia-like features are increasingly recognized in RA and can contribute to persistent pain independent of synovial inflammation [13]. In line with this concept, pain retained high centrality, whereas DAS28-CRP assumed a more peripheral position. This reinforces the need for comprehensive pain assessment and management beyond inflammation-focused treatment escalation [5,13].

Functional status and RA-specific quality of life (HAQ and RAQoL) demonstrated high centrality within the clinical network but did not show direct associations with UNs once shared variance was controlled. Their influence appears largely indirect, operating through pain intensity and global symptom perception. This aligns with reports of patient subgroups experiencing persistent pain and psychosocial burden despite adequate inflammatory control, emphasizing the multidimensional nature of RA impact [14,15].

The quality-of-life network provided complementary insights. UNs showed inverse associations with selected SF-36 domains, particularly Health Change and Role–Emotional functioning. Health Change reflects subjective appraisal of disease trajectory, suggesting that UNs may be especially sensitive to perceived lack of improvement. The association with emotional role limitations underscores the relevance of psychosocial functioning. Although emotional distress is clinically relevant in RA [16,17], DASS-42 did not occupy a central or intermediary position in the clinical network, indicating that discordance is not reducible to psychological distress alone but reflects broader experiential dimensions.

Taken together, UNs occupied distinct positions across networks: clinically anchored to pain and, within the broader quality-of-life context, linked to perceived health change and emotional functioning. This dual structure may help explain persistent patient–physician discordance and is consistent with difficult-to-treat RA frameworks, in which pharmacological optimization alone may be insufficient when non-inflammatory drivers dominate the patient experience [2,5].

Including healthy controls provided a contextual reference for the magnitude of impairment in the RA cohort. As expected, RA patients exhibited lower SF-36 scores and higher disability than controls, while overall psychological distress did not differ significantly, suggesting that UNs reflect disease-specific experiential burden rather than generalized distress. Although RA patients were older than controls, the direction and magnitude of group differences were clinically consistent with the established literature.

Several limitations warrant consideration. The cross-sectional design precludes causal inference, and network edges represent conditional associations rather than directional effects [11,18]. The sample size (*n* = 133), although sufficient for exploratory network modeling and supported by acceptable correlation stability coefficients, remains modest relative to the number of estimated parameters. Therefore, findings should be interpreted within an exploratory framework. In addition, the sample size precluded subgroup analyses (e.g., seropositive vs. seronegative RA or early vs. established disease), which may represent clinically relevant sources of heterogeneity and should be examined in future larger studies. UNs were defined exclusively through patient–physician discordance, which may also reflect communication gaps or expectation mismatches rather than strictly unmet therapeutic interventions. The single-center design further limits generalizability across different healthcare settings and patient populations. Future multicenter and longitudinal studies are warranted to validate these findings and to determine whether targeting central nodes such as pain modifies UNs over time.

From a clinical perspective, systematic assessment of pain mechanisms, emotional role functioning, and patient expectations may help identify UNs not captured by disease activity-driven decisions, thereby supporting a more integrative and patient-centered management approach.

## 5. Conclusions

In patients with rheumatoid arthritis, UNs—operationalized as the signed difference between patient and physician global assessments (ΔPGA–PhGA)—were not solely associated with inflammatory disease activity but were embedded within interconnected clinical and psychosocial domains. Network analysis identified pain as the most central clinical correlate of UN, whereas perceived health change and emotional role limitations characterized their position within the quality-of-life network.

These findings underscore the limitations of inflammation-focused strategies alone and highlight the importance of integrating patient-reported outcomes, pain mechanisms, and psychosocial dimensions into routine assessment. Rather than reflecting isolated deficits in disease control, UNs appear to emerge from multidimensional interactions between symptom burden, patient perceptions, and functional impact.

## Figures and Tables

**Table 1:** (**A**) Demographic and Clinical Characteristics of the Study Population. (**B**) Distribution of Unmet Needs (UN) in the Rheumatoid Arthritis Cohort (n = 133).

(A)				
Variable	HC (n = 57) Mean ± SD	RA (n = 133) Mean ± SD	Cohen’s d	*p* Value
Age (years)	44.84 ± 13.98	51.77 ± 12.24	−0.55	0.002
HAQ raw score (0–24)	1.55 ± 3.98	12.59 ± 11.72	−1.08	<0.001
DASS-42 total score	24.63 ± 18.31	27.57 ± 24.14	−0.13	0.768
RAQoL score	2.20 ± 3.65	11.16 ± 8.60	−1.18	<0.001
SF-36 Physical Functioning	81.94 ± 21.48	60.98 ± 27.49	0.81	<0.001
SF-36 Role–Physical	70.41 ± 33.72	43.23 ± 40.02	0.71	<0.001
SF-36 Role–Emotional	72.79 ± 35.14	51.88 ± 44.84	0.49	0.006
SF-36 Vitality	63.27 ± 22.93	50.71 ± 21.11	0.58	<0.001
SF-36 Mental Health	68.90 ± 21.73	61.92 ± 18.84	0.35	0.007
SF-36 Social Functioning	74.23 ± 22.16	62.22 ± 27.93	0.45	0.009
SF-36 Bodily Pain	74.69 ± 25.99	48.80 ± 27.47	0.96	<0.001
SF-36 General Health Perception	62.14 ± 16.80	43.57 ± 21.25	0.92	<0.001
SF-36 Health Change	58.67 ± 24.77	45.68 ± 28.12	0.48	0.003
(**B**)				
**Variable**		**Value**		
ΔPGA–PhGA (mean ± SD)		2.27 ± 2.05		
Median (IQR)		2.00 (1.00–4.00)		
Min–Max		−1 to 8		
High UN (≥2 units), n (%)		81 (60.9%)		

Abbreviations: RA, rheumatoid arthritis; HC, healthy control; HAQ, Health Assessment Questionnaire; DASS-42, Depression Anxiety Stress Scale-42; RAQoL, Rheumatoid Arthritis Quality of Life; SF-36, Short Form-36. ΔPGA–PhGA represents the signed difference between patient and physician global assessments. The median value of the RA cohort (2 units) was used as a data-driven threshold for descriptive categorization of higher UNs. Negative values of Cohen’s d indicate higher mean values in the RA group compared with healthy controls.

**Table 2:** Node Centrality Indices in the SF-36-Based Quality-of-Life Network (Strength, Signed Strength, Betweenness, and Closeness).

Node (Quality-of-Life Network)	Strength	Signed Strength	Betweenness	Closeness
Role Limitations due to Physical Problems (Role–Physical, SF-36)	1.1326	1.1326	0.1944	0.1316
Vitality (Energy/Fatigue, SF-36)	1.0248	1.0248	0.1111	0.1137
Social Functioning (SF-36)	0.9475	0.9475	0.0556	0.1223
Mental Health (SF-36)	0.9136	0.8949	0.0556	0.1105
Bodily Pain (SF-36)	0.8895	0.8895	0.1111	0.1245
Physical Functioning (SF-36)	0.8811	0.8811	0.0833	0.1349
General Health Perception (SF-36)	0.8557	0.8557	0.1944	0.1239
Role Limitations due to Emotional Problems (Role–Emotional, SF-36)	0.8104	0.5290	0.0833	0.1174
Health Change (Compared with One Year Ago, SF-36)	0.7859	0.4350	0.1111	0.1024
Unmet Needs (UNs)	0.3255	−0.3255	0.0278	0.0855

**Table 3:** Node Centrality Indices in the Clinical Network (Strength, Signed Strength, Betweenness, and Closeness).

Node (Clinical Network)	Strength	Signed Strength	Betweenness	Closeness
Pain Intensity (VAS)	1.330	1.330	0.400	0.154
Rheumatoid Arthritis Quality of Life (RAQoL)	1.315	1.165	0.700	0.177
Unmet Needs (UNs)	1.132	0.713	0.000	0.134
Disease Activity Score in 28 Joints (DAS28-CRP)	0.776	0.506	0.600	0.177
Functional Disability (Health Assessment Questionnaire raw score; HAQ)	0.730	0.730	0.000	0.145
Psychological Distress (Depression Anxiety Stress Scale-42; DASS-42)	0.511	0.511	0.000	0.134

## Data Availability

The datasets generated and/or analyzed during the current study are available from the corresponding author upon reasonable request.

## Abbreviations

The following abbreviations are used in this manuscript:

RA	Rheumatoid arthritis
UN	Unmet needs
PGA	Patient global assessment
PhGA	Physician global assessment
VAS	Visual analog scale
DAS28-CRP	Disease Activity Score in 28 joints with C-reactive protein
HAQ	Health Assessment Questionnaire
RAQoL	Rheumatoid Arthritis Quality of Life
DASS-42	Depression Anxiety Stress Scale-42
SF-36	Short Form-36
CS	Correlation stability
DMARDs	Disease-modifying antirheumatic drugs
csDMARDs	Conventional synthetic DMARDs
bDMARDs	Biologic DMARDs
tsDMARDs	Targeted synthetic DMARDs
HC	Healthy control
